# Frontal Headache and Swelling: A Case Report of Pott's Puffy Tumor

**DOI:** 10.7759/cureus.50670

**Published:** 2023-12-17

**Authors:** Kaltham Al Doaibel, Fatema Hasan, Hawra Antar, Zahra Hasan, Sharif Alfeki

**Affiliations:** 1 General Practice, Qatif Central Hospital, Qatif, SAU; 2 General Practice, Xi'an Jiaotong University, Xi'an, CHN; 3 General Practice, Wenzhou Medical University, Wenzhou, CHN; 4 General Practice, National University of Science and Technology, Muscat, OMN; 5 Emergency Medicine, Dallah Hospital, Riyadh, SAU

**Keywords:** craniotomy, subgaleal abscess, epidural abcess, frontal headache, acute sinusitis

## Abstract

Pott's puffy tumor, a rare complication of frontal sinusitis, poses a diagnostic challenge due to its infrequency and diverse clinical manifestations. Recognizing this condition promptly is crucial due to the potential for severe neurological compromise. We present the case of a 32-year-old male who presented with a one-week history of frontal headache, tenderness, and swelling following an upper respiratory tract infection. The physical examination revealed a tense, erythematous swelling over the frontal region. Laboratory results showed elevated inflammatory markers. Computed tomography revealed an epidural abscess secondary to frontal sinusitis. An emergent craniotomy was performed to evacuate the collection, followed by intravenous antibiotic therapy. The patient recovered with no neurological deficits. This case emphasizes the importance of considering Pott's puffy tumor in patients with frontal swelling and associated symptoms. Despite its rarity, a multidisciplinary approach involving imaging, microbiological analysis, and surgical intervention enables an accurate diagnosis and successful management. Timely recognition and appropriate treatment, including surgical drainage and targeted antibiotics, are critical for achieving favorable outcomes.

## Introduction

Pott's puffy tumor, a rare and potentially serious complication of frontal sinusitis, presents a diagnostic challenge due to its infrequency and diverse clinical manifestations [1]. Named after Sir Percivall Pott, an 18th-century English surgeon, this condition manifests as a subperiosteal abscess with associated osteomyelitis of the frontal bone, often leading to an epidural abscess [1,2]. The progression of the disease can result in significant neurological compromise, necessitating prompt recognition and intervention. While frontal sinusitis is a common entity, its progression to Pott's puffy tumor is exceptionally rare, emphasizing the importance of considering this entity in patients with persistent frontal headaches and swelling [3]. This case report describes the clinical course of a young male patient who presented with characteristic features of Pott's puffy tumor.

## Case presentation

A 32-year-old Saudi man presented to the emergency department of Dallah Hospital in Riyadh, Saudi Arabia, in February 2023, with a one-week history of progressive frontal headache, associated with localized tenderness and swelling over the forehead. The patient reported an initial upper respiratory tract infection two weeks prior, which had resolved spontaneously without medical intervention. He denied any recent trauma, sinusitis, dental infections, or systemic symptoms such as fever or chills. There was no history of immunodeficiency or intravenous drug use.

Upon physical examination, the patient exhibited a tense, erythematous swelling over the frontal region. The overlying skin was warm to the touch, and palpation revealed tenderness and fluctuation. Neurological examination demonstrated no focal deficits, and the patient had intact cranial nerve function. There were no signs of meningeal irritation.

Initial laboratory investigations were notable for an elevated white blood cell count of 15,000 cells/mm^3^, with a left shift. C-reactive protein was markedly elevated at 150 mg/L (Table 1). Blood cultures were obtained, and empiric antibiotic therapy with ceftriaxone and vancomycin was initiated.

**Table 1:** Initial laboratory investigations 'N' denotes normal values, 'L' signifies low values, and 'H' indicates high values.

Laboratory Test	Result	Reference Range	Indicator
Blood Glucose	100 mg/dL	70-100 mg/dL	N
Hemoglobin	13.5 g/dL	13.8-17.2 g/dL	N
Platelet Count	250,000 cells/mm³	150,000-450,000 cells/mm³	N
White Blood Cell Count	15,000 cells/mm³	4,000-11,000 cells/mm³	H
Erythrocyte Sedimentation Rate	40 mm/h	0-20 mm/h	H
C-reactive Protein	150 mg/L	0-5 mg/L	H
Serum Creatinine	0.8 mg/dL	0.6-1.2 mg/dL	N
Alanine Aminotransferase	25 U/L	7-56 U/L	N
Aspartate Aminotransferase	30 U/L	13-39 U/L	N

**Figure 1:**
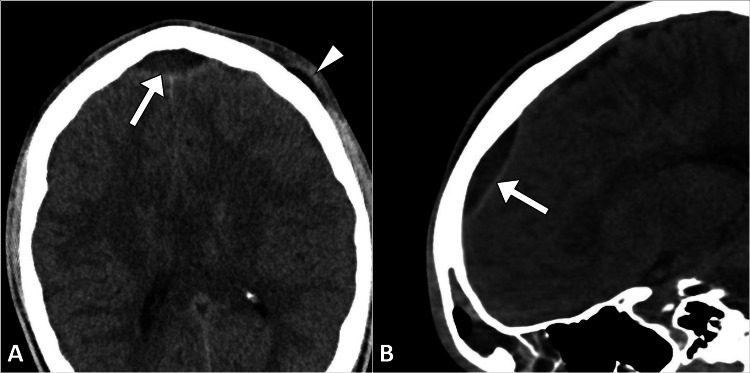
Axial (A) and sagittal (B) CT head images illustrate an epidural collection (arrow) in the frontal region, accompanied by a subgaleal collection (arrowhead) over the left frontal region. CT: computed tomography.

**Figure 2:**
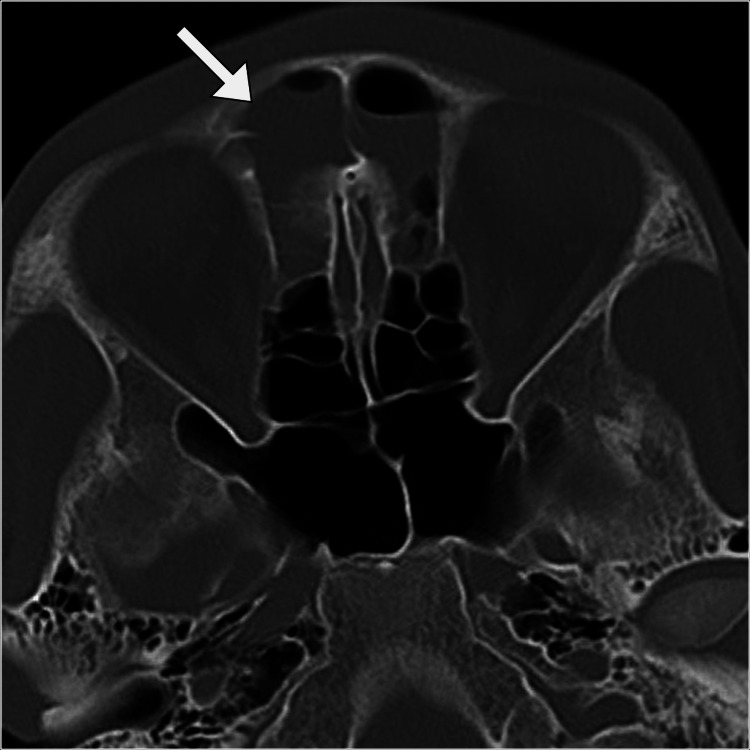
Axial CT head image displaying mucosal thickening with an air-fluid level in the frontal sinuses (arrow), indicative of acute sinusitis. CT: computed tomography.

The non-contrast computed tomography scan revealed a well-defined collection in the epidural space of the frontal region, in association with near-complete opacification of the frontal sinuses. Additionally, a small subgaleal left frontal collection was noted. These findings confirmed the suspicion of an epidural abscess secondary to frontal sinusitis (Figures 1, 2).

The patient underwent emergent craniotomy for evacuation of the epidural collection. Intraoperatively, purulent material was drained, and cultures confirmed *Staphylococcus aureus* as the causative organism. Broad-spectrum antibiotics were continued postoperatively, guided by sensitivity results. The patient's hospital course was complicated by a transient postoperative fever, which resolved with conservative management.

In the subsequent days, the patient demonstrated significant clinical improvement, with resolution of the frontal swelling and normalization of inflammatory markers. The patient commenced a four-week course of intravenous antibiotics, including ceftriaxone and vancomycin, guided by initial blood culture results. Following the intravenous phase, he was transitioned to oral therapy, receiving a two-week course of oral antibiotics, specifically cephalexin.

Laboratory monitoring during the antibiotic treatment showed a progressive decline in inflammatory markers, confirming a positive response to the chosen antibiotic regimen.

The patient was discharged after completing the six-week antibiotic course, showing sustained improvement and meeting discharge criteria. Follow-up assessments at subsequent intervals confirmed the absence of symptoms, indicating a successful resolution of Pott's puffy tumor.

## Discussion

The presented case of Pott's puffy tumor underscores the complex interplay between sinusitis and intracranial complications. Pott's puffy tumor is a rare manifestation of frontal sinusitis, and its prompt recognition is imperative for preventing severe neurological sequelae [1]. The diagnostic challenge lies in the subtle progression from frontal sinusitis to Pott's puffy tumor, necessitating a high index of suspicion. Frontal headaches and swelling, though common in sinusitis, should prompt a thorough evaluation, particularly in cases where initial conservative measures fail to provide relief [3].

Frontal sinusitis, the precursor to Pott's puffy tumor, is commonly initiated by bacterial or, less frequently, viral infections. The pathogens most frequently associated with Pott's puffy tumor include *Staphylococcus aureus* and *Streptococcus* species [4-6]. Several host factors play a role in predisposing individuals to Pott's puffy tumor. Anatomical variations in sinus anatomy, such as nasal polyps or structural abnormalities, can impede proper sinus drainage, creating an environment conducive to infection [3].

Radiological imaging plays a central role in the diagnostic process. Computed tomography scans are particularly valuable for visualizing the frontal sinus, identifying signs of sinusitis, and detecting the presence of subperiosteal abscesses [7]. Magnetic resonance imaging is another valuable tool, providing detailed soft tissue contrast and aiding in the assessment of the surrounding structures. Both imaging modalities contribute to a comprehensive understanding of the extent and nature of the pathology [2,7].

Pott's puffy tumor should be differentiated from other forehead swellings, including soft tissue abscesses, cysts, or neoplasms. The integration of clinical findings with imaging and microbiological data is crucial in ruling out alternative diagnoses and ensuring the accurate identification of Pott's puffy tumor [2].

Surgical intervention, exemplified by emergent craniotomy for abscess evacuation in our case, remains a cornerstone of management [7]. The decision for surgery was supported by the radiological findings of an epidural abscess compressing the frontal lobe. Long-term follow-up revealed no recurrence of symptoms and complete resolution of the epidural collection, supporting the efficacy of the chosen therapeutic approach. Antimicrobial therapy in these patients should be directed against both gram-positive and gram-negative bacterial species [4,5].

## Conclusions

In conclusion, this case report underscores the importance of considering Pott's puffy tumor as a potential diagnosis in patients presenting with frontal swelling and associated symptoms. The atypical manifestation of this rare condition, characterized by osteomyelitis of the frontal bone concurrent with sinusitis, poses a diagnostic challenge for clinicians.

Through a multidisciplinary approach involving radiological imaging, microbiological analysis, and surgical intervention, we successfully identified and managed the underlying cause in our patient. Timely recognition and appropriate treatment, including surgical drainage and targeted antibiotic therapy, played a pivotal role in achieving a favorable outcome. Furthermore, the long-term follow-up demonstrated no recurrence of symptoms, indicating a successful resolution of Pott's puffy tumor.
